# Electrochemical gating-induced reversible and drastic resistance switching in VO_2_ nanowires

**DOI:** 10.1038/srep17080

**Published:** 2015-11-20

**Authors:** Tsubasa Sasaki, Hiroki Ueda, Teruo Kanki, Hidekazu Tanaka

**Affiliations:** 1 Institute of Scientific and Industrial Research, Osaka University, 8-1 Mihogaoka, Ibaraki, Osaka, 567-0047, Japan

## Abstract

Reversible and drastic modulation of the transport properties in vanadium dioxide (VO_2_) nanowires by electric field-induced hydrogenation at room temperature was demonstrated using the nanogaps separated by humid air in field-effect transistors with planer-type gates (PG-FET). These PG-FETs allowed us to investigate behavior of revealed hydrogen intercalation and diffusion aspects with time and spatial evolutions in nanowires. These results show that air nanogaps can operate as an electrochemical reaction field, even in a gaseous atmosphere, and offer new directions to explore emerging functions for electronic and energy devices in oxides.

The transport characteristics of transition metal oxides are sensitive to redox reactions because the valence numbers of the transition metal ions are easily changed by the reactions, which affect the carrier density and/or stabilization of the crystal structure^1^,^2^,^3^,^4^. Of the prototypical materials, VO_2_ is promising as it undergoes a metal-insulator transition (MIT) and the resistance changes by orders of magnitude around 340 K. In VO_2_ nano to microstructures^5^,^6^,^7^,^8^,^9^,^10^,^11^,^12^,^13^, the coupling of the MIT with mechanical^7^,^8^,^9^, optical^13^, thermal^12^ and electronic properties^9^,^11^ can be used in tunable resonators, optical switchers, electronic and thermo-sensing devices. Furthermore, only a few atomic percent of the hydrogen- or oxygen-intercalation and -desorption in VO_2_ cause drastic changes in the transport properties, equal to that caused by inducing the MIT^14^,^15^,^16^,^17^,^18^,^19^,^20^. Conventionally, controlling the amount of hydrogen/oxygen ions in an oxide has been conducted by annealing the samples under redox gas atmospheres^1^,^4^,^15^,^16^, in an aqueous solution^17^,^18^ and with a hydrogen spillover method^14^,^19^. Every method requires high temperatures of at least 150 °C. Recently, on the other hand, an electric field has been shown to be a means of both hydrogenation and oxidization in oxides at room temperature^20^,^21^,^22^,^23^. For example, a strong electric field in ionic liquid (IL) gates induces oxygen vacancy formation in VO_2_^22^. In IL or CAN (amorphous 12CaO·7Al_2_O_3_ with a nanoporous structure) gates including water^21^,^24^, furthermore, hydrogen ions can be intercalated by positive electric field. Thus electrochemical gating including water electrolysis has a full potential to allow tuning of doping level at room temperature. In this paper, we report investigation of systematic transport modulation in VO_2_ nanowires by electrochemical gating *via* air gap under humid condition and establishment of the ion diffusion model, giving the hydrogen ion intercalation and diffusion aspects with time and spatial evolutions in VO_2_.

## Results

### Proposed device structure

**Figure 1:**
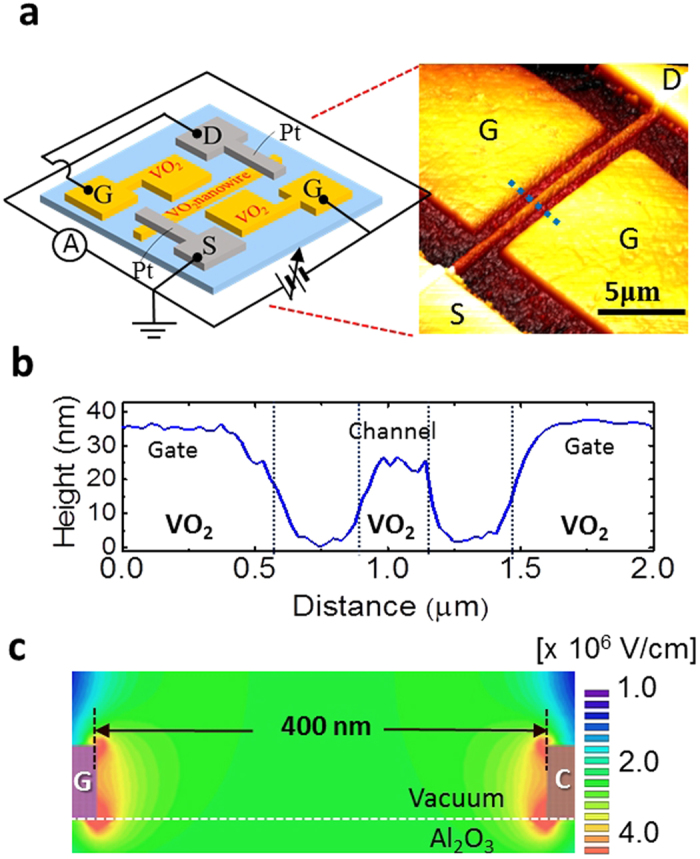
VO_2_ nanowire device with planer-type gates. (**a**) The typical device architecture and an atomic force microscope (AFM) image of the VO_2_ channel area. S, D and G indicate the source, drain and gate electrodes, respectively. (**b**) Cross-sectional AFM image, taken at the blue dashed line in (**a**). (**c**) Cross-sectional electric field map, determined using finite element analysis at *V*_G_ = 100 V through the 400-nm vacuum gap between G and the channel (**c**) on an Al_2_O_3_ substrate under vacuum.

As a proper device structure to perform this experiment, we have prepared planer-type field effect transistor with side gates and a nanoscaled wire channel separated by air nanogaps (PG-FET) illustrated in Fig. 1a. This type of device has following advantages: An electric field can be applied under various different gas and vapor atmospheres through the air nanogap. In this study, air with various humidity levels and dry air were used. Secondly, the electric-field induced ion intercalation and diffusion aspects with the time and spatial evolutions in the vicinity of interface can be systematically investigated because ions are intercalated from channel edges of both side and diffuse from the sides in VO_2_. Especially a narrower channel often enhances interface effect. An atomic force microscope image of a device is shown in Fig. 1a and the height profile is in Fig. 1b. The thicknesses of the channels and the gap distance were roughly 35 nm and 400 nm, respectively. The channel widths (*w*) were systematically changed from 3 μm to 400 nm (see Methods and Supplementary Information section A for details). Figure 1c shows a cross-sectional electric field-map, obtained using a Finite Element simulation using typical experimental parameters (see Supplementary Information section B). The electric field converged at the edges of the channels, providing the strongest effect on the electrochemical reactions. Thus the field-induced ion intercalation begins at the lateral sides of the nanowires

### Drastic resistance modulation by electrochemical induced-hydrogenation

**Figure 2:**
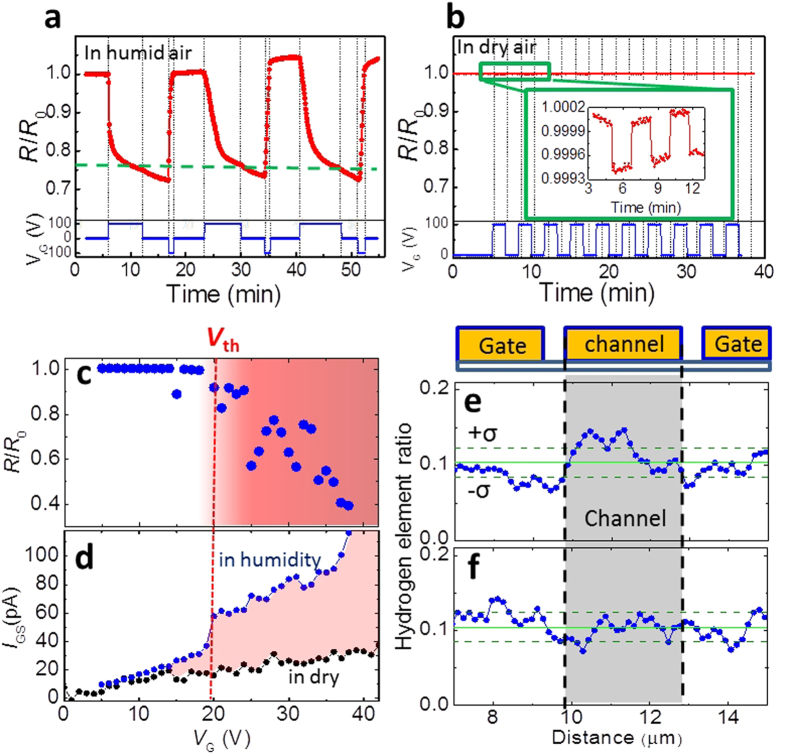
Effect of the electric field on the transport properties and hydrogen intercalations in a VO_2_ channel. (**a**,**b**) Time dependence of the normalized resistance (*R*/*R*_0_, where *R*_0_ is the pristine resistance of a non-treated VO_2_ channel at 300 K with applied *V*_G_ values of 100, 0 and −100 V) (**a**) in humid air and (**b)** in dry air. The green dashed line in (**a)** indicates the rough saturation of *R*/*R*_0_. The inset in (**b)** is a magnified view. (**c**) *V*_G_ dependence of *R*/*R*_0_ after applying a *V*_G_ for 20 minutes. (**d**) *V*_G_ dependence of the current between the gate and source electrodes (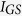) under a humidity of 60% (blue dotted-line) and in dry air (black line). (**e**,**f**) The relative elemental ratios for hydrogen normalized by oxygen (**e**) in a device after applying *V*_G_ = 100 V and (**f**) in a pristine device. The solid and dashed green lines represent the averages of the hydrogen atom profiles and the standard deviations, respectively.

Figure 2a shows the reversible and non-volatile resistance modulation in a VO_2_ nanowire channel (*w* = 500 nm), found by applying a positive and negative *V*_G_ at 300 K under a humidity of around 50%. The normalized resistance (*R*/*R*_0_), where *R* and *R*_0_ are the resistance and the pristine resistance before applying a *V*_G_ at 300 K, respectively, slowly continued to drop during the application of *V*_G_ = + 100 V down to the saturation line at roughly *R*/*R*_0_ = 0.75. This state was held after removal of the *V*_G_. Namely, it exhibited a non-volatile memory effect. In contrast, the resistance increased again at *V*_G_ = −100 V. Regarding slow resistive drops by an electric field, it is suggested that the origin would be related to mechanical relaxation or slow traps according to some reports^25^,^26^,^27^. In our PG-FET devices, on the other hand, such slow changes in the resistance were not observed under dry air condition (Fig. 2b). A steep resistance-switching of 0.06% occurred without any non-volatile memory effects, as shown in the inset of Fig. 2b. Approximately 10^12^ cm^−2^ electrons should be accumulated at V_G_ = 100 V and 400 nm-gap distance. From Hall measurements in VO_2_ thin films on Al_2_O_3_ substrates^28^, the carrier density was evaluated at ~4 × 10^18^ cm^−3^, equivalent to the Debye length of ~2 nm. Given modulation of resistivity in both lateral sides in our device geometry, the calculated modulation rate would be ideally 0.4%. Thus, it is considered that a pure carrier accumulation acts at lateral sides in dry air condition though the efficiency on the electrostatic effects was not so high compared with the ideal case. From the presence of the steep resistive switching in Fig. 2b, the origin of slow resistive drops under humid air conditions is not likely to attribute to the mechanical relaxation or slow traps as mentioned above, but rather to electrochemical reaction with intercalation of hydrogen ions^21^,^24^,^27^,^29^, which can significantly reduce resistivity in 3*d*-orbital sensitive systems^30^ (see Supplementary Information C for the intercalation (*V*_G_ = 100 V) and non-intercalation (*V*_G_ = 0 V) cases in detail) and/or color-switching properties known as electrochromism^31^. In VO_2_ intercalated hydrogen ions, a strong H-O bond induces electron transfer from hydrogen onto the oxygen atom, resulting in higher 3*d*-orbital occupancy of vanadium from V^4+^(3*d*^1^) to V^3+^(3*d*^2^)^5^. Figure 2c shows V_G_ dependence of *R*/*R*_0_ 20 minutes after applying the *V*_G_ to investigate the magnitude of resistance changes with variety of *V*_G_. The threshold voltage starting to the reduction (*V*_th_) was approximately 20 V and the magnitude of the resistance changes was enhanced with increasing *V*_G_. The resistance fluctuation in over 20 V would be due to fluctuation for level of humidity. The resistance modulation is sensitive to the level of humidity. In addition, repetitive intercalation and desorption of hydrogen ions may slightly change crystallinity of VO_2_ channels, causing the resistive fluctuation. In response with the resistance changes, the current between the gate and source electrodes (*I*_GS_) suddenly increased at around 20 V as seen in Fig. 2d, corresponding to the *V*_th_ in Fig. 2c. Subtracting the current recorded under humid conditions from that recorded under dry conditions gives the current generated by the electrolysis of water. Accordingly, the density of generated hydrogen ions increases with increasing *V*_G_ and the number of intercalated hydrogen ions in VO_2_ increases. Additionally, Fig. 2e shows the ratio of hydrogen elements in a device after applying *V*_G_ = 100 V, investigated by ToF-SIMS (time-of-flight secondary ion mass spectrometer). It can be indicated that hydrogen content in VO_2_ channel is higher than that in other area in a device after applying the *V*_G_, though the hydrogen content is roughly averaged because spatial resolution in the measurement is several hundred nanometer at most, whereas the hydrogen contents remain unchanged in a pristine device in Fig. 2f (see Supplementary Information section D in detail).

### Establishment in ion diffusion model by electrochemical gating

**Figure 3:**
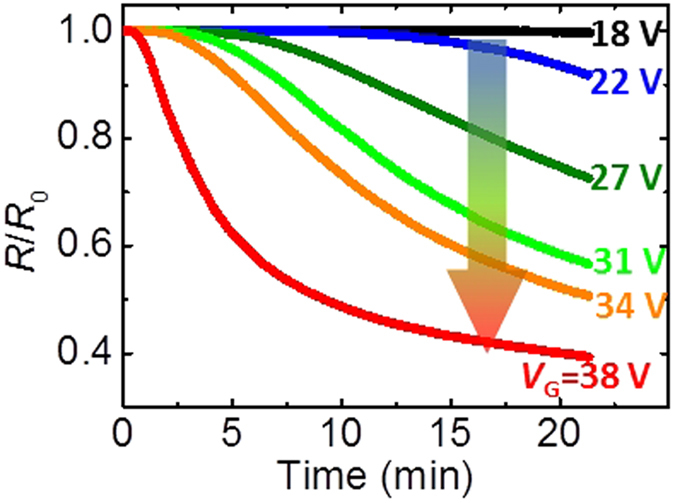
Transport properties from redox reactions in VO_2_ by the electrolysis of water with various *V*_G_ values. Time dependence of *R*/*R*_0_ for different *V*_G_ values under a humidity of 60% at 300 K.

It is known that a 1% hydrogen intercalation per VO_2_ unit cell induces almost one order of magnitude reduction in resistivity^17^,^18^. Based on the empirical facts and the intercalation from the channel edge by applying electric gate from lateral sides, the amount of intercalated hydrogen ions and the effect on the diffusive aspect in VO_2_ can be evaluated by investigating the resistive behavior with a variety of *V*_G_ values. Figure 3 shows the time dependence of the resistive modulations with various V_G_ under a humidity of 60%. The resistance began to decrease above *V*_G_ = 22 V, approximately corresponding to the *V*_th_. In the characteristic features, the magnitude of resistance reduction rate increases with increasing *V*_G_ and the initial reduction speed is faster in the larger *V*_G_, while the reduction speed became slower with time.

**Figure 4:**
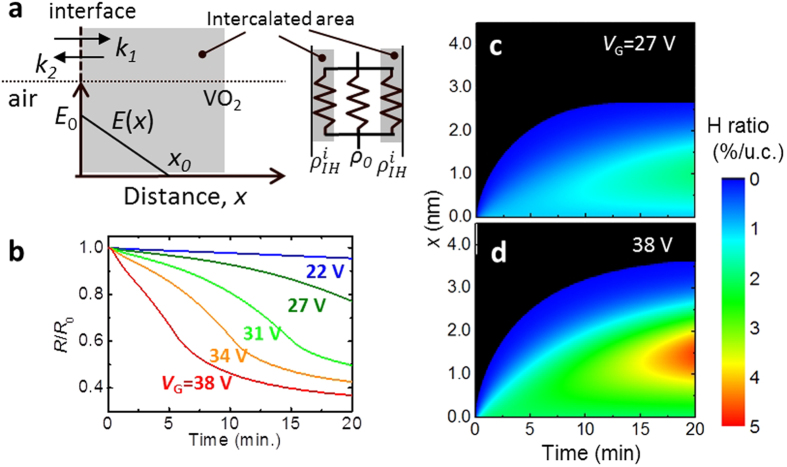
Simulated transport properties determined by the diffusion of intercalated ions. (**a**) Schematic of the ion intercalation at the interface between air and VO_2_, resulting from chemical kinetics and the diffusion area (gray region), found using Fick’s diffusion model under an electric field (*E*(*x*)) derived by Poisson’s equation. The simulated channel resistance was calculated using the parallel resistor model for the intercalated (*ρ*_IH_) and non-intercalated (*ρ*_0_) resistivities and by taking each width into consideration, as shown on the right side in (**a)**. (**b**) Time dependence of the simulated *R*/*R*_0_ with a variety of *V*_G_ values for 500 nm wide VO_2_ channels. (**c**,**d**) Spatial- and time-evolution maps of the hydrogen ion concentration at (**d**) *V*_G_ = 27 V and (**e**) 38 V. *x* = 0 indicates the interface.

To understand such transport behaviors in a variety of *V*_G_, theoretical investigations have been carried out with a combination of chemical reaction kinetics at the interface and ion diffusion model under an electric field^32^. The external hydrogen ions stochastically react with VO_2_ at the interface. The reaction rate depends on the external hydrogen ion concentration (*n*_H+_) generated by electrolysis of the absorbed water. The *n*_H+_ tends to increase with increasing current between the gate and channel electrodes (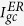), promoting formation of HVO_2_. While at the same time, desorption of hydrogen, namely return to VO_2_, would occur because of the natural recovery of resistivity by the thermal energy as seen in Supplementary Information section E. Thus assuming the reversible reaction, the time evolution-dependence of the concentration of intercalated ions inside VO_2_ at the interface (*n*_inter_) can be written as a differential equation with respect to time (*t*):




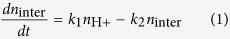




where *k*_1_ and *k*_2_ are the forward and reverse reaction rate constants, depending on the activation energy at the interface and temperature. Next, we consider how the intercalated ions diffuse in VO_2_. Theoretically, for ion diffusion, the ionic fluxes likely arise from the gradients of the ion concentration and the electric gradients in solid-state materials^32^. Thus, as *n*_HVO2_ is the hydrogen ion concentration in VO_2_, the hydrogen ion flux (*J*_HVO2_) can be described as: 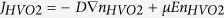, where *D* is the diffusivity, *μ* is the mobility and *E* is the internal electric field in VO_2_. The first and second terms represent ion diffusion by the ion concentration gradient and by an electric field, respectively. The *E* resulting from *V*_G_ is screened by mobile electrons in VO_2_ according to Poisson’s equation, given as a function of the distance (*x*) from the interface (*x* = 0), namely, 
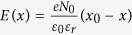
, where *e* is the elementary charge, *N*_0_ is the carrier density in VO_2_, and *ε*_r_ and *ε*_0_ are the relative permittivity of VO_2_ and the permittivity of a vacuum, respectively. *x*_0_ can be expressed as: 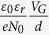 as a function of *V*_G_, where *d* is the distance between the gate and channel. *N*_0_ and *ε*_r_ change with depending on the magnitude of intercalated hydrogen ions, however, *N*_0_/*ε*_r_ can be treated as a constant value because the change rate of *ε*_r_ is roughly proportional to that of *N*_0_^33^. Thus, the length of *x*_0_ would be determined only by the magnitude of *V*_G_, and *E* linearly decreases as a function of *x* and become zero at *x*_0_, as shown in Fig. 4a. To conduct the unsteady state analysis, we use Fick’s second law in the one dimensional case, namely, 
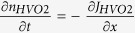
, which predicts the time and spatial evolutions of the ion concentration. With this the following equation was obtained:




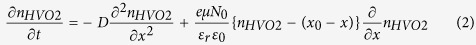




Furthermore, using a parallel resister model for the resistivity in the intercalated (*ρ*_IH_) and non-intercalated (*ρ*_0_) parts in Fig. 4a and assuming a 1% hydrogen intercalation per VO_2_ unit cell induces one order of magnitude reduction in the resistivity^17^,^18^, *R*/*R*_0_ could be evaluated as the following equation:




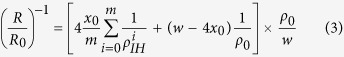




where 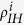 is the part of the spatially divided resistivity in the diffuse area taking 2 × _0_ in consideration of ion diffusion by the ion concentration gradient, which is divided into *i*, and is given as: 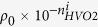, where 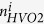 represents the hydrogen concentration in segment *i* (*i*: integral), derived using the finite difference method in equation (2) and the boundary conditions in equation (1). A more detailed derivation is outlined in the Supplementary Information section F.

The experimental results in Fig. 3 are significantly reproduced in Fig. 4b as *k*_1_, *k*_2_ and *μ* are fitting constants, though the more perfect reproduction requires more consideration efforts in the setting parameters, for example, considering *k*_1_ and *k*_2_ parameters depending on *V*_G_, which make enhanced the reduction rate of resistivity with increasing *V*_G_, and more precise resistive simulation like a random resistor network. Furthermore, this simulation enough includes the important essence of ion diffusion behavior, time and spatial evolutions of the ion diffuse in VO_2_ are shown in Fig. 4c,d, which are in the cases at *V*_G_ = 27 and 38 V, respectively, where *x* = 0 indicates a channel edge. Hydrogen ions expand with time and the concentration increase with increasing *V*_G_. Within the frame work of this model, interestingly, hydrogen ions accumulate in an inner area, clearly observed at *V*_G_ = 38 V in Fig. 4d. This is caused by the continuous non-equilibrium states of the ion intercalation and diffusion by an electric field. In more detail, this accumulation is induced by the slower ion-diffusion rate in the inner part, depending on deduction of internal electric field with increasing *x*. As a token of this scenario, following the removal of any *V*_G_, this accumulation fades away in time due to the ion-diffusion by concentration gradient and finally the concentration become homogeneous and equilibrium states. This diffusion behavior at *V*_G_ = 0 is significantly reproducible for the persisting resistance decrease even after removal of the *V*_G_ in Fig. 2a, shown by the simulation in Supplementary Information section G. Thus this device suggests to be a kind of proton pumps in solid-state system.

### Drastic resistance modulation in narrower nano-channel

**Figure 5:**
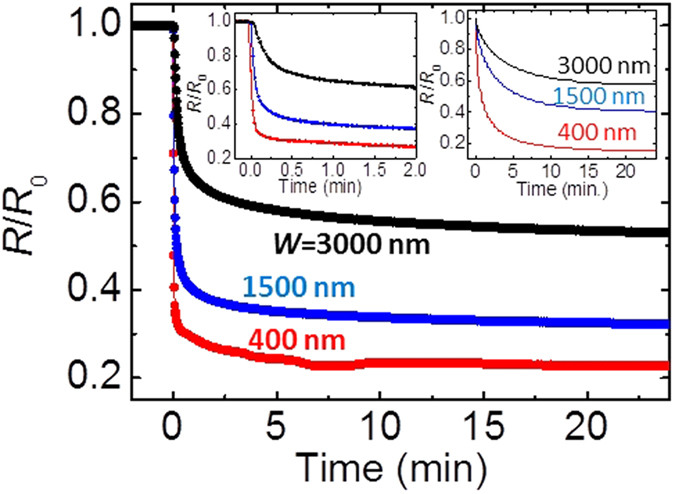
Enhancement of *R*/*R*_0_ with decreasing *w*. Time dependence of the simulated *R*/*R*_0_ values for 400 nm, 1500 nm and 3000 nm wide VO_2_ channels at *V*_G_ = 100 V. The left and right insets show the magnified view of Fig. 5 and the simulation results, respectively.

Moreover, this model predict that the magnitude of resistance reduction rate enhance with decreasing the channel width (*w*) because the effect on the interface diffusion due to the electrochemical gating from lateral sides become more prominent in narrower *w*. Figure 5 shows the time dependence of *R*/*R*_0_ with a variety of wire widths (*w* = 400, 1500 and 3000 nm) at *V*_G_ = 100 V and 300 K. The saturation values for *R*/*R*_0_ were enhanced with decreasing *w* and the sharpness of the resistance deduction in the initial process differs among the three devices as seen in the left inset of Fig. 5, magnified view from 0 to 2 minutes of Fig. 5. This behavior is in agreement with the simulation in the right inset of Fig. 5. Thus the further narrower channel will provide perfect electrochemical gating causing metal-insulator transition in whole channel area.

## Discussion

These results show that an air nanogap significantly works as an electrochemical reaction field, even in a gaseous atmosphere, and it is expected that the intercalated elements have an impact on reversibly changing in the physical properties of VO_2_. This interfacial effect was more enhanced in smaller nanoscaled channels. This offers a new way to both investigate the fundamental physical properties on the effect of intercalation and non-equilibrium ion diffusion for a wide range of materials and may lead to the realization of new gas-sensing, storage applications and also ion pumps in solid-state materials.

## Methods

### Thin film growth

35-nm-thick VO_2_ films were prepared on Al_2_O_3_(0001) single crystal substrates by pulsed laser deposition using an ArF excimer laser at 450 °C under an oxygen pressure of 1.0 Pa. Using X-ray diffraction measurements, it was confirmed that the films were *b*-axis-oriented without any impurity phases.

### Device fabrication

The films were patterned into nanowire channels with planer-type field gates by nanoimprint lithography and reactive ion etching using O_2_ and SF_6_ gases. As advantages of this method, we can easily obtain 200 nm to 400 nm-air gaps between VO_2_ gate electrodes and VO_2_ channels, and fabricate many PG-FETs at one process. Also, since the resistivity of VO_2_ is roughly 1 ohm cm even in insulating region at room temperature, VO_2_ gate should enough work as gate electrodes in electrostatic effect. Pt/Cr electrodes were deposited by radio-frequency sputtering. Ohmic contacts between the VO_2_ films and electrodes were confirmed.

### Electrical measurements

The transport characteristics were measured using a two terminal method with a Keithley 2635A. *V*_G_ was applied using a Keithley 236 and the currents between the gate and source electrodes were monitored simultaneously. The temperature of the device was controlled by a Peltier-based temperature stage (T95, Linkam). The gaseous conditions, from dry air to 80% humidity were controlled in a glovebox. The humidity fluctuation was within ±2%.

## Additional Information

**How to cite this article**: Sasaki, T. *et al.* Electrochemical gating-induced reversible and drastic resistance switching in VO_2_ nanowires. *Sci. Rep.* **5**, 17080; doi: 10.1038/srep17080 (2015).

## Supplementary Material

Supplementary Information
