# Supplementary material for: Electrochemical gating-induced reversible and drastic resistance switching in VO2 nanowires
Source: Sci Rep. 2015 Nov 20;5:17080. doi: 10.1038/srep17080 (PMC4653652; doi:10.1038/srep17080)
Supplement: Supplementary Information [file srep17080-s1.pdf]

# SUPPLEMENTARY INFORMATION

## Electrochemical gating-induced reversible and drastic resistance switching in $VO_2$ nanowires

Tsubasa Sasaki, Hiroki Ueda, Teruo Kanki* and Hidekazu Tanaka*

*Institute of Scientific and Industrial Research, Osaka University, 8-1 Mihogaoka, Ibaraki, Osaka 567-0047, Japan*

**Section A. Sample preparation and pictures of devices.**

$VO_2$ nanowire or microwire channels (*w*=400 nm to 3 μm) and $VO_2$ gates with 400-nm gaps were prepared using nano-imprint lithography. After patterning, Pt/Cr electrodes were patterned by photolithography. Using this technique, 64 nano-devices were fabricated at the same time on a 4.0-mm × 4.0-mm area, as shown in Fig S1a and b.

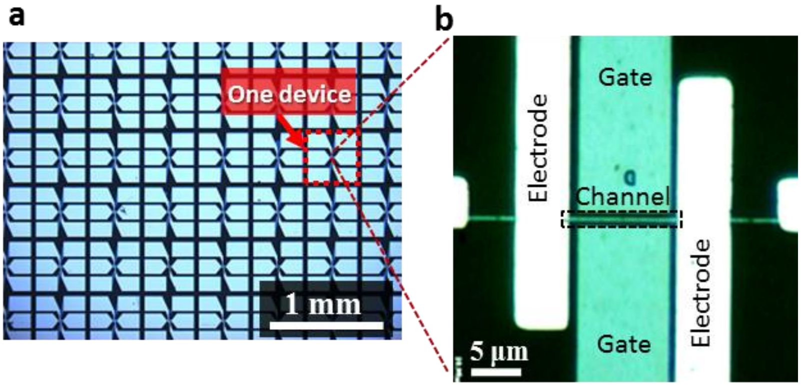


**Figure S1: $VO_2$-based planer-gate-type electric field transistor.**
**a** and **b**, Optical micrographs of the devices over a wide area (**a**) and a magnification of part of a device (**b**).

**Section B. Static Electric Field Analysis using Finite Element Simulations**

To analyze electric field distribution in the devices, numerical simulations were performed with a Finite Element Method using AMaze (Advanced Science Laboratory, Inc.). Then a three dimensional device geometry was assumed, as shown in Fig. S2a. Figure S2b shows cross-sectional potential images at the center of a device, forming the basis of Fig. 1c.

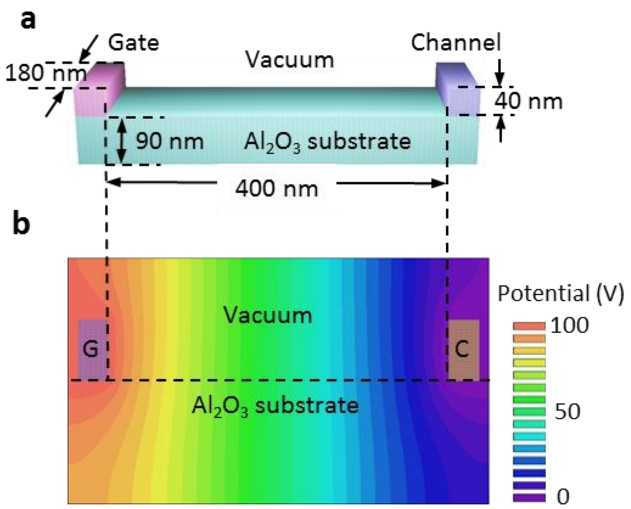


**Figure S2: Device geometry used for the static-electric field simulation.**
**a**, The device area of ($x$, $y$, $z$)=(500 nm, 200 nm, 200 nm), which was divided as: (d$x$, d$y$, d$z$)=(6 nm, 2.5 nm, 2.5 nm). The relative permittivity of $Al_2O_3$ is 8.5. The potential was 100 V at the gate and the channel was grounded. **b**, Cross-sectional potential map with $V_G$=100 V.

## Section C. Resistive behavior with applied $V_G$=100 V and without $V_G$(=0 V) in 60 % humid air

Figure S3 shows time dependence of resistive behavior with $V_G$=100 V and 0 V in humid air condition of 60 %. We confirmed that the resistance in $V_G$=0 V didn't change, while that in $V_G$=100 V drastically reduced in the initial few minutes and gradually continue to decrease.

**Figure S3:** Time dependence of resistance with and without $V_G$ (100 V and 0 V) in humid air condition.

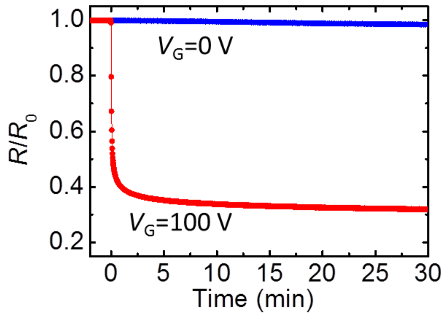


## Section D. Cross-sectional elemental mapping by Tof-SIMS

To investigate the ratios of the V, O and H atoms, ToF-SIMS (time-of-flight secondary ion mass spectrometer) measurements were conducted by a technician at the Foundation for Promotion of Material Science and Technology of Japan (MST). Figure S4b–s shows the negative ion images related to V, O, H, Al and Pt, respectively, for a device with $V_G$=100 V. A pristine device with an area of approximately 37 μm × 37 μm, is shown in Fig. S4a. The spatial resolution and step were roughly 300 nm and 150 nm, respectively.

In the detailed analysis on the relative elemental concentrations, the cross-sectional data across the channel were sampled, indicated by the diagonal red line in Fig. S4a.

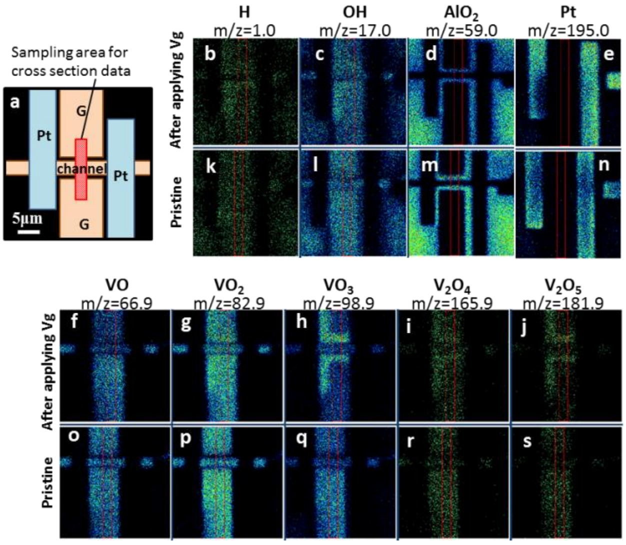

**Figure S4: ToF-SIMS negative ion images of a variety of ions related to V, O, H, Al and Pt. a**, Schematic of a device measured using Tof-SIMS. **b** and **k** are images for H; **c** and **l** are for OH; **d** and **m** are for $AlO_2$; **e** and **n** are for Pt; **f** and **o** are for VO; **g** and **p** are for $VO_2$; **h** and **q** are for $VO_3$; **i** and **r** are for $V_2O_4$; and **j** and **s** are for $V_2O_5$ in devices after applying $V_G$=100 V and a pristine device, respectively.

Figure S5a, b, e and f shows the intensities of each element in a device after applying $V_G$=100 V (a and b) and in a pristine device (e and f). Figure S5c, d, g and h shows the normalized ratios between V, H and O in a device after applying $V_G$=100 V (c and d) and a pristine device (g and h). From this it was identified that H increased in the channel after $V_G$=100 V was applied, over the standard deviation (+σ) (Fig. S5d). However, it remained almost unchanged in the pristine device (Fig. S5h).

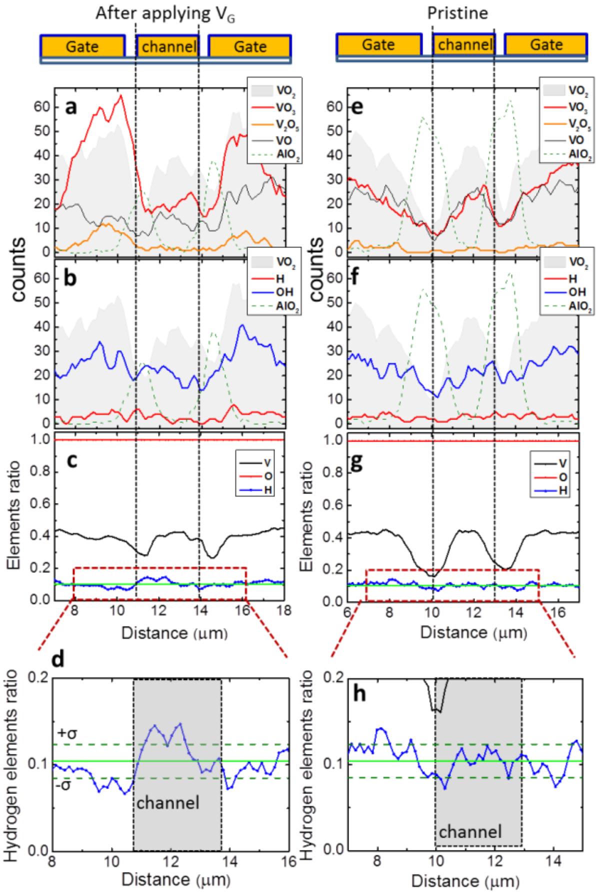

**Figure S5: Cross-sectional ion maps of the images in Fig S4. a**, **b**, **e** and **f**, Cross-sectional ion maps for $VO_2$, $VO_3$, $V_2O_5$, VO and $AlO_2$ in devices after applying $V_G$=100 V and for a pristine device, respectively. **c**, **d**, **g** and **h**, The relative elemental rates for V and H, normalized by O in a device after applying $V_G$=100 V and for a pristine device, respectively. The solid and dashed green lines in **d** and **h** represent the averages of the H atom profiles and the standard deviations (σ), respectively.

**Section E. The natural recovery of resistance by the thermal energy**

Figure S6 shows the resistance behavior in $VO_2$ nanowires ($w$=500 nm) as a function of temperature. In the resistive measurement without any $V_G$, the resistances between the before and after values correspond to point "A" in the inset of Figure S6. 20 minutes after applying a $V_G$ of 100 V the resistance had reduced approximately by half, to "B" in Figure S6. This resistive state remained even following the removal of any $V_G$, which agrees with the behavior in Figure 2a. Thereafter, as the temperature increased to 380 K over the transition temperature without $V_G$, the resistance decreased accompanied by the MIT. In this process, hydrogen ions are more easily diffusible and removable from $VO_2$ channel with increasing temperature[17,18,21]. When the temperature returns to 290 K, the suppressed resistive state was recovered to its original state at point "A" in Figure S6.

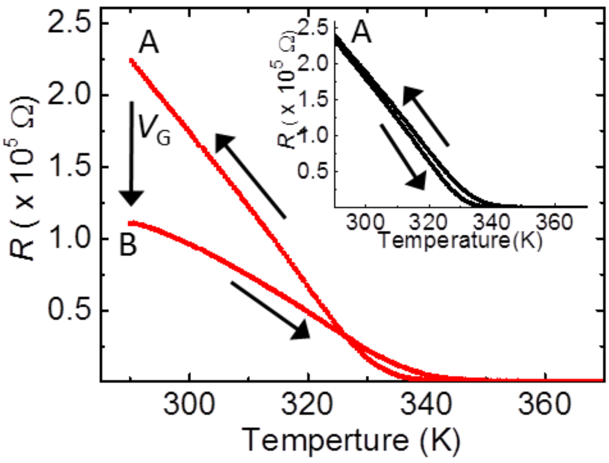


**Figure S6:** Temperature dependence of resistance of a $VO_2$ channel at $V_G$=0 V after applying $V_G$=100 V for 20 minutes at 290 K under a humidity of 50%. The inset shows the regular temperature vs resistance without $V_G$. The pristine resistance at 290 K is located at point "A"

**Section F. Details on the calculations used to obtain Fig 4c and d.**

To evaluate the time and spatial evolution of the hydrogen ion concentration in $VO_2$ according to equations (1) and (2), numerical analysis with the finite difference method was carried out using Mathematica 9.0. Before the calculations, equation (2) was changed to

$$\frac{\partial n_{HVO2}^{i}}{\partial t} = D\,\frac{n_{HVO2}^{i+1} - 2n_{HVO2}^{i} + n_{HVO2}^{i-1}}{\Delta x^2} + \frac{e\mu N_0}{\varepsilon_r \varepsilon_0}\left\{\left(\frac{x_C}{\Delta x} - i\right) n_{HVO2}^{i-1} + \left(1 + i - \frac{x_C}{\Delta x}\right) n_{HVO2}^{i}\right\}$$

, with following transformations; $\frac{\partial n_{HVO2}(x,t)}{\partial x} = \frac{n_{HVO2}(x,t) - n_{HVO2}(x-\Delta x,t)}{\Delta x}$ , and $\frac{\partial^2 n_{HVO2}(x,t)}{\partial x^2} =$

$\frac{n_{HVO2}(x+\Delta x,t)-2n_{HVO2}(x,t)-n_{HVO2}(x-\Delta x,t)}{\Delta x^2}$. Then, $x$ was replaced with $i\Delta x$, where $n_{HVO2}^{i}$ and $n_{HVO2}^{i-1}$ represent $n_{HVO2}((i\text{-}1)\Delta x,t)$ and $n_{HVO2}(i\Delta x,t)$, respectively. Thus, the numerical calculations could be performed using $e$=1.602×10$^{-19}$ C and $\varepsilon_0$=8.859×10$^{-12}$. $n_{H+} = I_{gc}^{ER} \times \frac{\alpha}{S_{inter}} = 0.574V_G^2 \times \frac{\alpha}{S_{inter}}$ was obtained from Fig. S8, where $S_{inter}$ is the number of $VO_2$ unit cells at the interface, written as: $S_{inter}$=2.691×10$^{6}$, which is the cross-sectional area where $V_G$ was applied (35 nm × 10000 nm) divided by the volume of a $VO_2$ unit cell (0.4554 nm × 0.4554 nm × 0.2856 nm), $\alpha$=3281 (which is a constant value that includes the intercalated rate), $N_0$=1.0×10$^{26}$ m$^{-3}$ [s1, s2], $\varepsilon_r$=36[s3] and $E_0$=2.5 × 10$^{6}V_G$ V/m at the interface using the experimental parameters, and $k_1$=0.001, $k_2$=0.050 and $\mu$=0.045 in the fitting constants from Fig 3 and Fig. 5. It was assumed that the state was completely metallic when over 2.25% of the hydrogen ions were intercalated in the region, because the resistivity in the samples underwent a change of approximately 2.25 orders of magnitude between the metallic and the insulating states at room temperature. The simulated curves in Fig 4b and c were derived using equation (3) and $\rho_{IH}^{i} = \rho_0 \times 10^{-n_{HVO2}^{i}}$. Figure S7 shows the results calculated at $V_G$=38 V. For this calculation, the equations were obtained from $n_{HVO2}^{0}(t)$ to $n_{HVO2}^{30}(t)$ as a function of time.

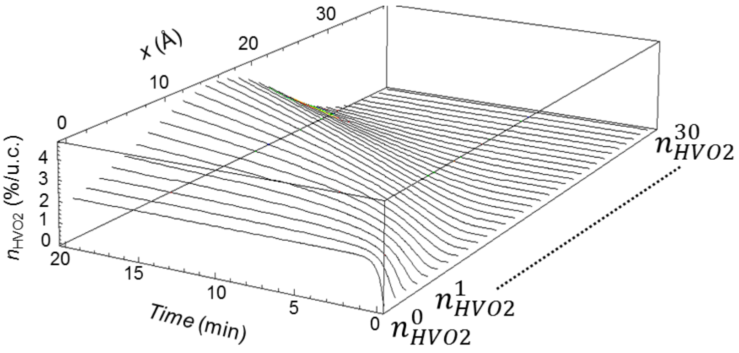


**Figure S7:** Simulations for the time-evolution in each $n_{HVO2}^{i}$ ($i$=0 to 30) at $V_G$=38 V.

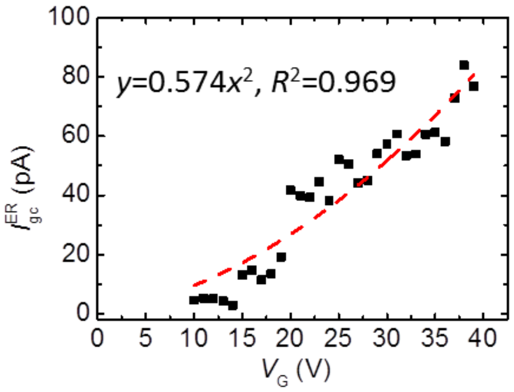


**Figure S8:** Current generated by the electrolysis of the adsorbed water ($I_{gc}^{ER}$) as a function of $V_G$.

**Section G. Reproduction for the persisting resistance decrease after removal of the $V_G$ in Fig.2a by this simulation.**

This simulation can reproduce the persisting resistance decrease after removal of the $V_G$. Figure S9a shows resistance drops with time at $V_G$=100 V until 10 min. and at $V_G$=0 V over 10 min. Even following the removal of $V_G$ the resistance continuously decreases. This behavior is due to the ion diffusion effect by the first term for concentration gradient in equation (2), though the second term can be eliminated because of $V_G$=0. The simulated diffusion behavior is seen in Figure S9b.

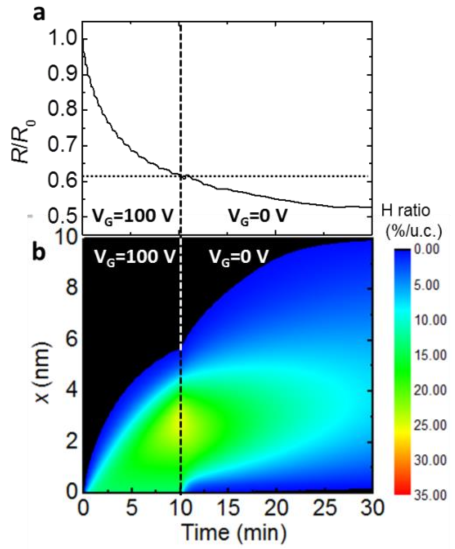


**Figure S9:** Time dependence of **a,** resistance and **b,** hydrogen ion diffusion behaviors at $V_G$=100 V and 0 V.

## References


s1. Kim, H.-T.; Chae, B.-G.; Youn, D.-H. Maeng, S.-L.; Kim, G.; Kang, K.-Y.; Lim, Y.-S. *New J. Phys* **2004**, 6, 52.

s2. Ruzmetov, D.; Heiman, D.; Claflin, B., Narayanamurti, V.; Ramanathan, S. *Phys. Rev. B* **2009**, 79, 153107.

s3. Yang, Z.; Ko, C., Balakrishnan, V.; Gopalakrishnan, G.; Ramanathan, S. *Phys. Rev. B* **2010**, 82, 205101.
